# Supplementary material for: Transcriptional and Metabolic Response of a Strain of Escherichia coli PTS− to a Perturbation of the Energetic Level by Modification of [ATP]/[ADP] Ratio
Source: BioTech (Basel). 2024 Apr 10;13(2):10. doi: 10.3390/biotech13020010 (PMC11036233; doi:10.3390/biotech13020010)
Supplement: Supplementary file 1 [file biotech-13-00010-s001.zip › biotech-2939222-supplementary.pdf]

Supplementary Materials: Transcriptional and Metabolic Response of a Strain of *Escherichia coli* PTS⁻ to a Perturbation of the Energetic Level by Modification of [ATP]/[ADP] Ratio

Table S1. Primer sets used for RT–qPCR assays.

Pathway, group of genes or cellular process	Primer name[1]	Sequence
**ATP synthase**		
atpA	atpAa	5′ TGCACCAATCGACGGTAAAG 3′
	atpAb	5′ TGCACCAATCGACGGTAAAG 3′
atpD	atpDa	5′ GACCTCGAACACCCGATTGA 3′
	atpDb	5′ TCACCGATCTCGCCTTTCAT 3′
atpG	atpGa	5′ ATGCGCAAAGTGATTGGTCA 3′
	atpGb	5′ GACACCACCAGGTAGCCCAC 3′
atpI	atpIa	5′ CCTGGACATTCGCATTGG 3′
	atpIb	5′ GATCAGCGGCAAGAATACCG 3′
**Glucose transport**		
ompF	ompFa	5′ TTCGCGGGTCTTAAATACGC 3′
	ompFb	5′ AATTCTGGCAGCATATCGGTG 3′
galP	galPa	5′ CATGTATTACGCGCCGAAAA 3′
	galPb	5′ TGGCAAGTACGTTGGTCAGG 3′
ptsH	ptsHa	5′ TGACTTCCAACGGCAAAAGC 3′
	ptshb	5′ TTCGCCTTCTGCGGAGATAG 3′
ptsG	ptsGa	5′ AAAAGCGACAGGTACCAGCG 3′
	ptsGb	5′ CGCGCAGACGGGTAATACAT 3′
**Acetate transport**		
actP (*yjcG*)	actPa	5′ ATCACTGGCTTCCAGAACGG 3′
	actPb	5′ CCGTCATAGCCGGAGGTAAAC 3′
**Glycolysis and gluconeogenesis**		
glk	glka	5′ GAAGCGGTCATTCGCGTTTA 3′
	glkb	5′ GAAGCGGTCATTCGCGTTTA 3′
pgi	pgia	5′ ACTAACGGTCAGCACGCGTT 3′
	pgib	5′ TCAGAGAGCGGGTTATGGGT 3′
pgk	pgka	5′ TGGACAGGGTTTCGTCGTCT 3′
	pgkb	5′ AGATTACCTCGACGGCGTTG 3′
fbaA	fbaAa	5′ GGAAATCGAACTGGGTTGCA 3′
	fbaAb	5′ CGTAATCAACGTCTTCCGGC 3′
fbaB	fbaBa	5′ GTACAACACCGGGCGTCTG 3′
	fbaBb	5′ GCGGGTTAGCAGCAAATGAA 3′
fbp	fbpAa	5′ AAACAGGTTGCGGCAGGTTA 3′
	fbpAb	5′ CCGAGCGAAGGATCGTAAGT 3′

gapA	gapAa	5′ GGCTCCGCTGGCTAAAGTTA 3′
	gapAb	5′ GGCCATCAACGGTTTTCTGA 3′
eno	enoa	5′ GTTTCGTCGGTATGGCAGCT 3′
	enob	5′ GCCTTTACCCAGGAAACGG 3′
gpmA	gpmAa	5′ AGGCGTAAGCGAAGCAAAAG 3′
	gpmAb	5′ GGGTATGGATAGCGCGTTTC 3′
gpmB	gpmBa	5′-GGTATTGCACTGGGATGCCT-3′
	gpmBb	5′TAATCCACGCGCGAAATAGA 3′
pykA	pykAa	5′ CGTTACCACGTTAGGCCCAG 3′
	pykAb	5′ GCGAGCCGTGAGAAAAGTTC 3
pfkA	pfkAa	5′ CCATGTAGGAACCGTCACCG 3′
	pfkAb	5′ GTTGGCGGATGAAAATGTCC 3′
ppc	ppca	5′CAGAAATCACCGTCAGCAGC 3′
	ppcb	5′ CATAATGCGACGCCAGCTCT 3′
tpiA	tpiA	5′ AACTCCGGCTCAGGCACAG 3′
	tpiAb	5′ AGCCGCCGTACTGAATGATC 3′
**Pentose phosphate pathway**		
eda	edaa	5′ ATCCGTGCTATCGCCAAAGA 3′
	edab	5′ AACTGTGCACCCGCTTCAGT 3′
gnd	gnda	5′ GATCGGCGTAGTCGGTATGG 3′
	gndb	5′ TCTTCTCACGGGAACGGTTG 3′
rpiA	rpiAa	5′ GATGGGCGGCACTTCAGTAT 3′
	rpiAb	5′ GCCTTTCATTGTACCGAGCG 3′
rpiB	rpiBa	5′ GAGAGGTTGATGGCGGGATT 3′
	rpiBb	5′ AGGTTCGCTACAGACGACCG 3′
talA	talAa	5′ CTCAAAATCGTACCCGGTCG 3′
	talAb	5′ TACAAGTCCACCAGATGGCG 3′
talB	talBa	5′ ACCGTAGTGGCCGACACTG 3′
	talBb	5′ GGAATCTGCGCTGCGTTAAG 3′
tktB	tktBa	5′ CCCGAAAAGACCTTGCCAAT 3′
	tktBb	5′ AATATCAGCCATGCCCATCG 3′
edd	edda	5′ GTACCGCTGATGGCACGTCT 3′
	eddb	5′ GCTTTGAGCAGTTCACGCAC 3′
zwf	zwfa	5′ GCACGCGTAGTCATGGAGAA 3′
	zwfb	5′ CGGTAAACCTGGCACTCCTC 3′
**TCA and glyoxylate shunt**		
aceB	aceBa	5′ GAACTGGCTTTCACAAGGCC 3′
	aceBb	5′ TGTGGCGTAAAATGCGTCAC 3′
aceA	aceAa	5′ ACATGGGCGGCAAAGTTTTA 3′
	aceAb	5′ AACCAGCAGGGTTGGAACG 3′
aceK	aceKa	5′ GCGTTATCAGCGACCTACCG 3′
	acekb	5′ GTTGTCTGTCCCCAGCGTTT 3′
aceE	aceEa	5′ CGTGAAGAAGGTGTTGAGCG 3′
	aceEb	5′ TTGCTGATACCTGTGCCTGC 3′
aceF	aceFa	5′ GTCGTATCCTGCGCGAAGAC 3′
	aceFb	5′ CAGCATGCCAGGGATACCAC 3′
acnB	acnBa	5′ CCTGGTGTTGTTGGTCCGAT 3′
	acnBb	5′ TTACGCGAAGAACCCGTACC 3′

fumA	fumAa	5′ ATGTCGATCAACTGCAAGCG 3′
	fumAb	5′ GAAGCCGCCGTGTTTTTTAC 3′
fumC	fumCa	5′ CCCTAACGACGACGTGAACA 3′
	fumCb	5′ GAGGAATGAGTTGCTTGCGC 3′
fumB	fumBa	5′ GTACCCTCGGTACTGCAGCC 3′
	fumBb	5′ AGCGCTTGCTAACTTGACGG 3′
glcB	glcBa	5′ CTCCAGCACAGTTTGTCGGTT 3′
	glcBb	5′ ATTGGCATCGATTTGCAGCT 3′
icdA	icdAa	5′GACCGAAGCGGCTGACTTAA 3′
	icdaB	5′ GCAGTTTAGCGCCATCCATC 3′
lpd	lpda	5′ GGTGGTGCGATTGTCGGTAC 3′
	lpdb	5′ TGGATGGTCAGTGCGATGTC 3′
mdh	mdha	5′ CGGGTCTGCAACCCTGTCTA 3′
	mdhb	5′ CGTAGGCACATTCGACAACG 3′
sdhC	sdhCa	5′ TGGCGTATCACGTCGTCGTA 3′
	sdhCb	5′ AAAGGAGATTTTGGCGGAGC 3′
sdhB	sdhBa	5′ TGAACGGCAAGAATGGTCTG 3′
	sdhBb	5′ GATCACCGGTAAACCTGGCA 3′
sucA	sucAa	5′ GCGGCAAAGAAACCATGAAA 3′
	sucAb	5′ TTCGGTGCTGGTAATGTGCA 3′
sucB	sucBa	5′ GCAGTACGGTGAAGCGTTTG 3′
	sucBb	5′ CTTCCGGGTAACGTTTCAGG 3′
sucC	sucCa	5′ CCAAAATCTTCATGGGCCTG 3′
	sucCb	5′ GCAAATCAGATCGCCCTGTT 3′
**Anaplerotic genes**		
pckA	pckAa	5′ ACATGTTTATTCGCCCGAGC 3′
	pcbAb	5′ CTGTTCTTTCCACTGCGGGT 3′
maeB	maeBa	5′ TGGTTTGCGATTCAAAAGGC 3′
	maeBb	5′ GAGGGTACGTTTGCCGTCAT 3′
maeA	maeAa	5′ TGGTTTGCGATTCAAAAGGC 3′
	maeAb	5′ GAGGGTACGTTTGCCGTCAT 3′
ppsA	ppsAa	5′ TCAGCAGGAAACCTTCCTCAA 3′
	ppsAb	5′ GATAAGAGATGGCGCGATCG 3′
**Respiratory chain**		
cyoD	cyoDa	5′ CCTGGCAATGGCAGTGGTAC 3′
	cyoDb	5′ TGAAGACAAACGCCGTCATG 3′
cyoE	cyoEa	5′ GTGATCGGCTACTGTGCGGT 3′
	cyoEb	5′ GCGATGGCATAGGAGTGAGG 3′
frdB	frdBa	5′ TTGAGGTGGTGCGCTATAACC 3′
	frdBb	5′ GCCCAGCGCATCCAGTAAT 3′
frdD	frdDa	5′ TGGTCGCGTATTCCTGTTCC 3′
	frdDb	5′ CCGCAGGTACGTGGATTTTC 3′
napA	napAa	5′ GATGGGCTGCTATGACGACA 3′
	napAb	5′ GGTTAGTGATGCGCGACCA 3′
narG	narGa	5′ CGATTATCCGGCGACTTACG 3′
	narGb	5′ GCGAGCCGTGAGAAAAGTTC 3′
ndh	ndha	5′ GTCGATCGTAACCACAGCCA 3′
	ndhb	5′ GCATGGGCCAGATAGCTCAA 3′

nuoA	nuoAa	5′ CTGGTGGCCATGTTCTTCGT 3′
	nuoAb	5′ GCTTCCACAAAGCCTACCCA 3′
nuoB	nuoBa	5′ CGTTTTGGCGCAGAAGTATTG 3′
	nuoBb	5′ AGACGCTGAATAACCGGTGC 3′
nuoF	nuoFa	5′ TATCCGTACTCCCGAAACGC 3′
	nuoFb	5′ CGCCTTCGTAACCGTTTTTG 3′
nuoM	nuoMa	5′ CGGTAAAACGCGTATCACGG 3′
	nuoMb	5′ AGTGAACAAAAACCAGCGCC 3′
nuoN	nuoNa	5′ TGTCGCGTTGGGTAAAAACC3′
	nuoNb	5′ GAGAGAGTTTGAAGCCGAGGC 3′
ubiE	ubiEa	5′ GGCAGAATCCATCCGTATGC 3′
	ubiEb	5′ CCCCTGCCGTCAGATTGTAG 3′
**Fermentation and acetate production and utilization**		
ackA	ackAa	5′ CTGGTTCTGAACTGCGGTAGTTC 3′
	ackAb	5′ GGCAGGTGGAAACATTCGG 3′
acs	acsa	5′ GTGCGTAAAGAGATTGGCCC 3′
	acsb	5′ CGCAGAATACGGCGCATAAT 3′
ldhA	ldhAa	5′ GGCGTGATGATCGTCAATACC 3′
	ldhAb	5′ ACGTCCATACCCAACGAACC 3′
pflD	pflDa	5′ AAAGTCCGCGCTCGCTTAAT 3′
	pfldB	5′ TCTTTGCAGTAGTGGCGCAA 3′
pflB	pflBa	5′ AAGGTTCCTGCAAAGCGTACA 3′
	pflBb	5′ GTAAACGTCGAACACGCCCT 3′
poxB	poxBa	5′ AAAGTCCGCGCTCGCTTAAT 3′
	poxBb	5′ TCTTTGCAGTAGTGGCGCAA 3′
**Sigma factors**		
rpoA	rpoAa	5′ TCAACCTGAAAGGGCTGGC 3′
	rpoAb	5′ GGTGATATCGGCTGCAGTCA 3′
rpoC	rpoCa	5′ GGCGTTGAAGTGACCCAGAC 3′
	rpoCb	5′ GACGGCAGCGATTTCAGG 3′
rpoD	rpoDa	5′ GATTCTGCGACCACCGAAAG 3′
	rpoDb	5′ TCGATACCGAAACGCATACG 3′
rpoE	rpoEa	5′ GAACTATTGAGTCCCTCCCGG 3′
	rpoEb	5′ CGGACAATCCATGATAGCGG 3′
rpoH	rpoHa	5′ CAGTTGGCAACCTGGATTCC 3′
	rpoHb	5′ GCCATGGTAATGCAGCTTTTC 3′
porN	rpoNa	5′ TGAAACCGATGGTACTGGCC 3′
	rpoNb	5′ GCCTCGTGGACTATGCAGGT 3′
rpoS	rpoSa	5′ GGACGCGACTCAGCTTTACC 3′
	rpoSb	5′ CGACATCTCCACGCAGTGC 3′
rpoZ	rpoZa	5′ TGGCACGCGTAACTGTTCA 3′
	rpoZb	5′ TCCGCCTACCTGCATCTGAC 3′
**Regulators**		
arcA	arcAa	5′ ATCACCAAACCGTTCAACCC 3′
	arcAb	5′ ACGCTACGACGTTCTTCGCT 3′
arcB	arcBa	5′ AATCTGACGGCGCAGGATAA 3′

	arcBb	5′ TGACCCAGCTGTTGCAGATG 3′
cra (*fruR*)	craa	5′ TCTTGTGATCCCCGATCTGG 3′
	crab	5′ AGCAGGCAATCAGCAGTTGA 3′
cyaA	cyaAa	5′ AGCGCCAATTGCTACAACGT 3′
	cyaAb	5′ ACGGAAGCGGTTTTCATCAA 3′
glcC	glcCa	5′ TCGCCCTATTTGCGAAGTG 3′
	glcCb	5′ CACACAGTCGACGTTCCGAG 3′
iclR	iclRa	5′ CTTTATGGTCGGCAGCAGCT 3′
	iclRb	5′ ATTGACCGTTTCGCCAGACT 3′
ihfA	ihfAa	5′ GGCGAACAGGTGAAACTCTCTG 3′
	ihfAb	5′ GTAATGGGAATATCCTCGCCC 3′
fadR	fadRa	5′ CGCTGGGCTTCTACCACAAA 3′
	fadRb	5′ AATCTCGCCACTCTCATGCC 3′
fnr	fnra	5′ CGGAAAAGCGAATTATACGGC 3′
	fnrb	5′ TTCGTTGAGTGTGAACGGGA 3′
narL	narLa	5′ TGATTGACGATCACCCGATG 3′
	narLb	5′ ACCCTGTTCGCCATTACTCG 3′
**Stress**		
rsd	rsda	5′ TGGCATTAAGCCTGGCAAAG 3′
	rsdb	5′ TGAAATGTCCGGCAGACAAG 3′
soxR	soxRa	5′ AACAGCTTTCGTCCCAATGG 3′
	soxRb	5′ AAGGCAGCCACAACCAATACA 3′
soxS	soxSa	5′ CGATTACATTCGCCAACGC 3′
	soxSb	5′ GCGAGACATAACCCAGGTCC 3′
spoT	spoTa	5′ ATCCGATCTCTTCCCGGATG 3′
	spoTb	5′ GCACTGCATAAGCGAAGTCG 3′
